# The Medical Laws of Alabama

**Published:** 1888-07

**Authors:** Jerome Cochran

**Affiliations:** Senior Censor, M. A. S. A.


### THE MEDICAL LAWS OF ALABAMA.

---

*Editors Atlanta Medical and Surgical Journal:*

In compliance with your request I proceed to give you a sketch of the laws of Alabama for the regulation of the practice of medicine and the measure of success that has attended their administration.

In brief, then, under our law no one can begin the practice of medicine in this State until he has passed a successful examination before some one of the authorized boards of medical examiners established by the Act of the General Assembly. The authorized boards of medical examiners are the Board of Censors of the State Medical Association, which is known as the State Board, and of the Board of Censors of the county medical societies holding charters from the State Medical Association. There are sixty-six of these county societies, and therefore sixty-six county boards of medical examiners.

The standard of qualifications, the subjects and methods of examination, and the rules for the government of the examining boards, are such as may be from time to time prescribed by the State Medical Association.

The rules at present in force may be briefly summarized as follows:

1. All examinations must be in writing, and must comprise ten different branches, namely, chemistry, anatomy, physiology, natural history and diagnosis of diseases, physical diagnosis, surgery, mechanism of labor, obstetric operations, hygene and medical jurisprudence. Materia medica and the practice of medicine are intentionally omitted from this schedule.

2. The examinations are conducted by a paid supervisor, who cannot be a member of the board, in such way as to make consultation of books or persons impossible. The answers to the questions are separately valued from one hundred, which indicates a perfect answer, down acccording to the judgment of the examiners, and for a successful result the final average of the values of the answers must reach seventy-five. The time consumed in an examination is usually from six to ten days. There is an oral examination also, but this is not valued. It serves to clear up doubts and to give the examiners some general ideas as to the ability of the applicant.

3. Every written examination made by a county board is sent up to the State Board, is reviewed by them, and their opinion of it is transmitted in annual reports to the State Association. Neither the State Board, nor the State Association can reverse the decision of a county board; but if the association is not satisfied with the way any county board does their work a reprimand or a censure will secure better work in future.

4. The county boards examine none but graduates of reputable medical colleges; non-graduates are examined only by the State Board. All examinations are bound and kept on file, so that they can be produced if needed for testimony in the courts, or for any other purpose.

5. If any applicant believes that he has not been fairly treated by any county board, he can appeal to the State Board, which gives him a new examination. If any applicant is rejected by any county board, he cannot have another examination by any county board until after the lapse of twelve months.

There are other rules, but these are the principal ones, and will serve to show the general character of our system. For some years now the rejections have averaged one-fifth—twenty per centum of the applicants—all college graduates.

The strong point of our system—that which secures and guarantees its efficiency—is the supervision of the State Medical Association. Any one not familiar with our work would very naturally suppose that its weak point would be found in the large number of county boards. But our county boards give us strength in another way: in practice we find it easy to hold them up to quite a sufficiently high standard; and, at the worst, they cannot fall below the standard of the medical colleges.

It may be freely granted that if thoroughness of examination was the only thing to be considered, this could be better accomplished through a single State board. But thoroughness of examination is not the only thing we have to consider. Indeed, strange as it may seem, it is not even the principal thing we have to consider. Our great aim is the organization and discipline of the medical profession throughout the State; and the most potent of all the factors we are able to invoke in the accomplishment of this object grows out of the fact that the county medical societies, through their boards of censors, have been made the agents of the State for the administration of the law to regulate the practice of medicine. But for this more than half the county societies would never have been organized at all, while under the influence of this incentive we are able to maintain a county society in every county in the State.

Eclectics and homœpaths must pass the same examinations as regulars. Our law, indeed, does not recognize sectarian differences amongst doctors. With us, so far as the law goes, a doctor is simply a doctor. The State knows nothing about regulars, eclectics or homœpaths, but requires the same standard of qualifications for all. After an irregular doctor has passed our examination, and so demonstrated his fitness to practice medicine, if he desires to join his county society the way is open to him. By that act he ceases to be an irregular, and pledges himself to abide by the ethics of the American Medical Association, since the endorsement of the code of ethics is incorporated into the constitution of every one of our county societies. If a properly qualified graduate of an eclectic or of a homœpathic medical college, desires to array himself in the ranks of regular and legitimate medicine, we think it is wise to encourage him to do so, and we do not think it either wise or just to give him the cold shoulder. We have very few homœpathic practitioners in Alabama, but a considerable number of doctors who graduated in eclectic schools have availed themselves of the advantages we have to offer them and have become good, working members of our organizations.

We regard our law as almost ideally perfect. If the State would invite us to change it according to our wishes, we would not know what change to suggest. All we have to ask of the State is simply to let our law stand as it is and enforce it in the courts. I have the honor to remain

Very truly,

JEROME COCHRAN, M. D.,
Senior Censor, M. A. S. A.

